# A more detailed park prescription: How does landscape support human health behavior?

**DOI:** 10.3389/fpubh.2025.1696097

**Published:** 2025-12-15

**Authors:** Binyi Liu, Wanyue Lyu, Yuting Yin

**Affiliations:** 1 Faculty of Humanities and Art, Macau University of Science and Technology, Macau, China; 2 College of Architecture and Urban Planning, Tongji University, Shanghai, China; 3 School of Design, East China Normal University, Shanghai, China; 4 Shanghai Key Laboratory of Urban Regeneration and Spatial Optimization Technology, Shanghai, China

**Keywords:** urban parks, health behavior, landscape characteristics, plot-based evaluation, crowdsourced data

## Abstract

**Introduction:**

The role of urban parks in promoting public health is continuously evolving. For time-pressed urban residents, knowing the specific locations within parks that support various health behaviors can help them use these spaces more effectively for their daily usage. This study attempts to develop an analytical framework at the plot scale, using a 30*30 m grid as the basic unit, to assess the intensity of health behaviors driven by different landscape characteristics within parks.

**Methods:**

To investigate these relationships, seven representative urban parks in Shanghai were selected, with 68 standard-sized plots established as sampling sites. Firstly, computer vision-based semantic segmentation was employed to measure the landscape features within these spots. This was combined with systematic behavioral observation and coding to quantify the intensity of three types of health behaviors—exercise, leisure, and social activities—at each measurement spot. Subsequently, regression analysis was used to construct a model defining the relationship between landscape characteristics and health behavior intensity. This model was then applied to predict and visualize the intensity of health behaviors across the entire park area.

**Results:**

Results show that sky visibility, pavement coverage, and the presence of rough or uneven ground are significant predictors of the overall intensity of health-related behaviors in the studied plots. More specifically, trees, pavements, rough or uneven ground and resting facilities are closely associated with the intensity of exercise behavior, while shrubs primarily affect the intensity of leisurely behaviors. Mapping the intensity of health-related behaviors in Fuxing Park revealed that spaces for leisurely activities overlap with those for the other two types of health-related activities, whereas exercise and social behaviors exhibit spatial exclusivity.

**Discussion:**

Ultimately, the resulting visualizations, which map the distribution and intensity of different health behaviors, thereby serve a dual purpose. Firstly, they enable users to promptly locate areas within parks that are suitable for specific health-promoting activities, thereby helping to prevent potential conflicts between different types of behaviors. Secondly, by establishing clear relationships between plot-level landscape features and observed health behaviors, the framework provides park managers with an evidence-based tool for optimizing the allocation of environmental resources to support diverse recreational needs.

## 1 Introduction

The health benefits of natural environment exposure are well-documented, operating through key mechanisms including harm reduction, restorative processes, and capacity building (1–3). The critical role of urban green infrastructure in supporting public health resilience was powerfully underscored during the COVID-19 pandemic (4, 5). This recognition has paralleled the growing integration of nature-based interventions, such as park prescriptions, into health and social policy frameworks worldwide (6–8), providing structured channels for employing nature in preventive and promotional health strategies. A central challenge, however, lies in translating this evidence base into practice; significant variation in effective doses, suitable activities, and contextual success factors reveals a pressing need for standardized implementation and evaluation protocols to achieve scalability (9).

As the most accessible natural spaces within the built environment, parks function as critical nodes of GI for promoting urban resident health in high-density cities. Consequently, rigorous evaluation of the health benefits derived from parks is essential for optimizing their design and management and for informing user engagement. Notwithstanding, current research on urban park health benefits exhibits significant limitations. Planning-oriented research emphasizes meso-scale factors such as vegetation coverage, park provision, and spatial accessibility, yet demonstrates poor alignment with actual health behavior patterns (10–12). There is a growing body of research indicating that equitable access to urban green spaces can help reduce health disparities (13–15). However, without considering the specific needs of different social demographic groups (e.g., age, gender) and their interactions with distinct park environments, equitable access to quality green spaces may be compromised (15). Design-focused studies predominantly correlate landscape elements with health outcomes, often neglecting the specific local environmental features that facilitate health-promoting activities (16–18). Management-scale investigations typically yield data on usage frequency and duration but lack a feature-based analytical framework to address users’ specific health needs (19, 20). To bridge these gaps, this study proposes a plot-scale measurement and analysis approach. Integrating semantic segmentation with behavioral observation methods, this approach enables the systematic assessment, visualization, and prediction of intra-park health benefit variations. Furthermore, it provides actionable insights for design and management enhancements, alongside targeted health behavior recommendations, to augment the health-promoting functionality of urban parks.

## 2 Literature review

### 2.1 Health-promoting behaviors fostered through routine park visitation

Healthy behavior refers to individuals’ engagement in activities aimed at maintaining or enhancing their health status while mitigating health risks (21). Urban parks (UPs) function as critical venues that facilitate diverse health-promoting activities—including walking, jogging, gardening, mindfulness practices, and social interaction—thereby contributing to measurable improvements in residents’ physical and mental well-being. Health behaviors within these spaces are typically classified through complementary taxonomies. First, activity-based categorization delineates physical exercise (e.g., running, jogging, team sports), social interaction (e.g., picnicking, conversational gatherings, singing), and leisure relaxation (e.g., resting, meditation, reflective activities) (22–24). Second, intensity-based classification distinguishes sedentary, low-intensity, and moderate-to-vigorous intensity activities (25–27). Additional frameworks further differentiate active versus passive behaviors according to participant intentionality and objectives (28). This multidimensional taxonomy enables systematic identification of heterogeneous health behaviors across parks, providing foundational support for granular assessment of associated health benefits.

Health outcome emerges from dynamic transactions between natural environments and human behavioral patterns (9, 29). Optimizing spatial configurations to amplify opportunities for health-promoting behaviors constitutes a critical pathway for health enhancement (30, 31). This perspective coheres with established health behavior models and intervention frameworks, which underscore the triadic reciprocity among environments, behaviors, and health outcomes (32, 33). Consequently, health behaviors function as essential mediating pathways linking park exposure to health benefits (34), serving as vital metrics for assessing UP efficacy (35). Micro-scale evaluations of UP health contributions have employed mixed method approaches to operationalize health behaviors (27), utilizing indicators such as visitor volume and density (36, 37), visit frequency (11, 29), and duration of engagement (11). Illustratively, Cohen et al. (38) quantified park health impacts through longitudinal tracking of healthy behavior participation rates post-intervention, while Akpinar et al. (29, 39) derived public health metrics from physical activity frequency-duration composites. Nevertheless, such aggregate measures of generic usage obscure critical variations in spatially embedded behavioral characteristics across discrete health behavior typologies. Emerging evidence posits activity-specific intensity metrics as proxies for overall healthy behavior levels within parks. This approach has been operationalized in an empirical study by classifying user activities into light, moderate, and vigorous intensity strata to assess differential health outcomes across park typologies (40). Parallel methodology was applied to park trail systems, where aggregate health stimulus was quantified through spatially averaged intensity profiles of walking, running, and cycling (41). Concurrently, scholarly attention has extended to healthy behavior diversity as a mediator between environmental attributes and health outcomes. This construct has been performed through activity type enumeration, exemplified by Wang et al.’s behavioral inventory methodology (42).

Nevertheless, prevailing methodologies exhibit two fundamental limitations: (1) Predominant evaluation of UPs as undifferentiated wholes (41), resulting in inadequate characterization of intra-site spatial heterogeneity in health behavior manifestations; (2) Reliance on composite metrics—exemplified by Cohen et al.’s seminal proposal that aggregate park activity intensity effectively proxies health benefits (43)—which obscures typological differentiations. Crucially, disaggregating activity-specific intensities through geospatial mapping would generate granular behavioral intelligence. Such spatially explicit differentiation enables precision-targeted interventions for park design and management optimization, but existing plot-scale investigations remain constrained to examining singular dimensions (e.g., behavior diversity or intensity) in isolation when correlating environmental attributes with health behaviors. These approaches fail to account for spatial interdependencies between site-specific areas and the park-wide system, nor do they extrapolate such relationships into generalizable principles for evidence-informed planning and design in analogous contexts.

### 2.2 Environmental facilitators of health-promoting behaviors in park settings

The health-promoting capacity of UP is mediated through three interdependent environmental dimensions: planning distribution, functional configuration, and design quality. At the planning scale, visitation propensity serves as a primary behavioral indicator, modulated by spatial configuration attributes including service area magnitude (44, 45), facility density (46, 47), and accessibility metrics (44, 45, 48). Functional typologies—recreational zones, natural habitats, and sports facilities—fundamentally structure behavioral patterns by channeling activity modalities, with empirical evidence demonstrating fitness zones sustaining behavioral consistency, lawn/court complexes maximizing diversity, and pavilion clusters achieving peak density yet minimal heterogeneity (49).

Within the design quality paradigm, environmental preference theory provides the principal foundations for understanding how spatial affordances (e.g., McCormack’s cardinal dimensions: functional legibility, safety performance, esthetic valence, destination capital) and auxiliary moderators (hygienic maintenance regimes, spatial morphology indices, microclimatic regulation, biodiversity metrics) collectively modulate health behaviors (45, 50–52). Crucially, the perception-behavior nexus operates as a parallel mechanism wherein subjective constructs (perceived safety, maintenance quality, scenic attractiveness) mediate behavioral intentionality—exhibiting non-linear dynamics evidenced by counterintuitive findings such as negative associations between perceived cleanliness and vigorous activity despite overall quality’s positive correlation with Metabolic Equivalent of Task (MET)-minutes (53). This multidimensional framework substantiates UP as a complex socio-ecological system requiring integrated assessment approaches. Complementarily, affordance theory conceptualizes park design elements as behavioral catalysts that activate health-promoting practices through spatial provocations (54). Empirical investigations delineate specific environmental affordances—including pedestrian circuits (55), fitness infrastructure (22, 39, 56), restorative seating clusters (22, 56), phytogeographical configurations (39, 55), and convivial gathering nodes (39, 55)—that scaffold distinct behavioral repertoires. Crucially, spatial-behavioral congruence manifests through differentiated element-function pairings. For example, arboreal canopies optimize thermal microclimates for moderate-vigorous physical activity domains (jogging/aerobics/cycling) through shade mediation (57); unobstructed lawns facilitate sedentary socialization via prospect-refuge dynamics (58); biophilic assemblages induce restorative behaviors through attention restoration (51); purpose-built facilities scaffold structured exercise via task-specific provocation (59); while panoramic vistas enhance social co-presence through visual permeability (60). This affordance-based typology demonstrates how differentiated environmental elements directly modulate health behavior patterns through biomechanical, psychological, and social pathways.

Current research on environmental determinants of health behaviors in UP exhibits critical limitations in spatial granularity and inadequate consideration of intra-site heterogeneity. At the planning-functional level, investigations predominantly focus on meso-scale urban factors—such as aggregate park provision, spatial accessibility metrics, or internal functional zoning—which lack resolution to discern nuanced behavioral variations at actual usage scales. Design-oriented studies frequently correlate holistic landscape quality with generalized behavioral patterns, neglecting to account for locally specific environmental features that directly catalyze or constrain particular health activities. While emerging scholarship acknowledges differential behavioral catalysis by distinct landscape elements, methodological reliance on researcher-dependent qualitative assessments and unstructured observational approaches persists. These limitations impede the development of standardized quantification frameworks in UPs capable of systematically deconstructing element-behavior relationships and also, generating evidence-based guidelines for spatially optimized behavioral facilitation.

### 2.3 The aim of this study

Addressing extant methodological limitations, this study proposes a plot-scale analytical framework integrating computer vision-based semantic segmentation for quantifiable landscape measurement, systematic behavioral observation and coding for activity-intensity classification, multivariate regression modeling for spatially explicit environment-behavior linkage establishment, and crowdsourced geospatial extrapolation to predict domain-wide behavioral intensity gradients. This integrative approach systematically evaluates how landscape factors influence health behavior promotion, producing predictive hotspot visualizations based on affordance theory. It provides evidence-based, precision strategies for urban parks by synthesizing design and management principles to optimize resource allocation. Additionally, this research establishes a foundation for developing personalized health interventions adapted to specific park environments, enabling the public to more effectively use these spaces for health-promoting activities.

## 3 Materials and methods

### 3.1 Research sites

This study selected Shanghai as the case study city. As one of China’s highest-density cities, Shanghai was among the earliest in the country to establish urban parks. With a long tradition of research and practice in urban park development, it serves as a benchmark for park construction in megacities. According to data from the Shanghai Greening and City Appearance Administration, the city had a total of 973 parks as of the end of 2024. Among them, 512 are urban parks, which include comprehensive parks, specialized parks, historic famous parks, and community parks. As the number of parks in Shanghai continues to rise, enhancing the quality of existing parks, enriching their service functions, and improving park management have become crucial to efficiently utilizing parks in order to boost public health and well-being. One important aspect is to provide clear instructions on what health benefits park visitors may obtain in different areas within the parks.

**Figure 1:**
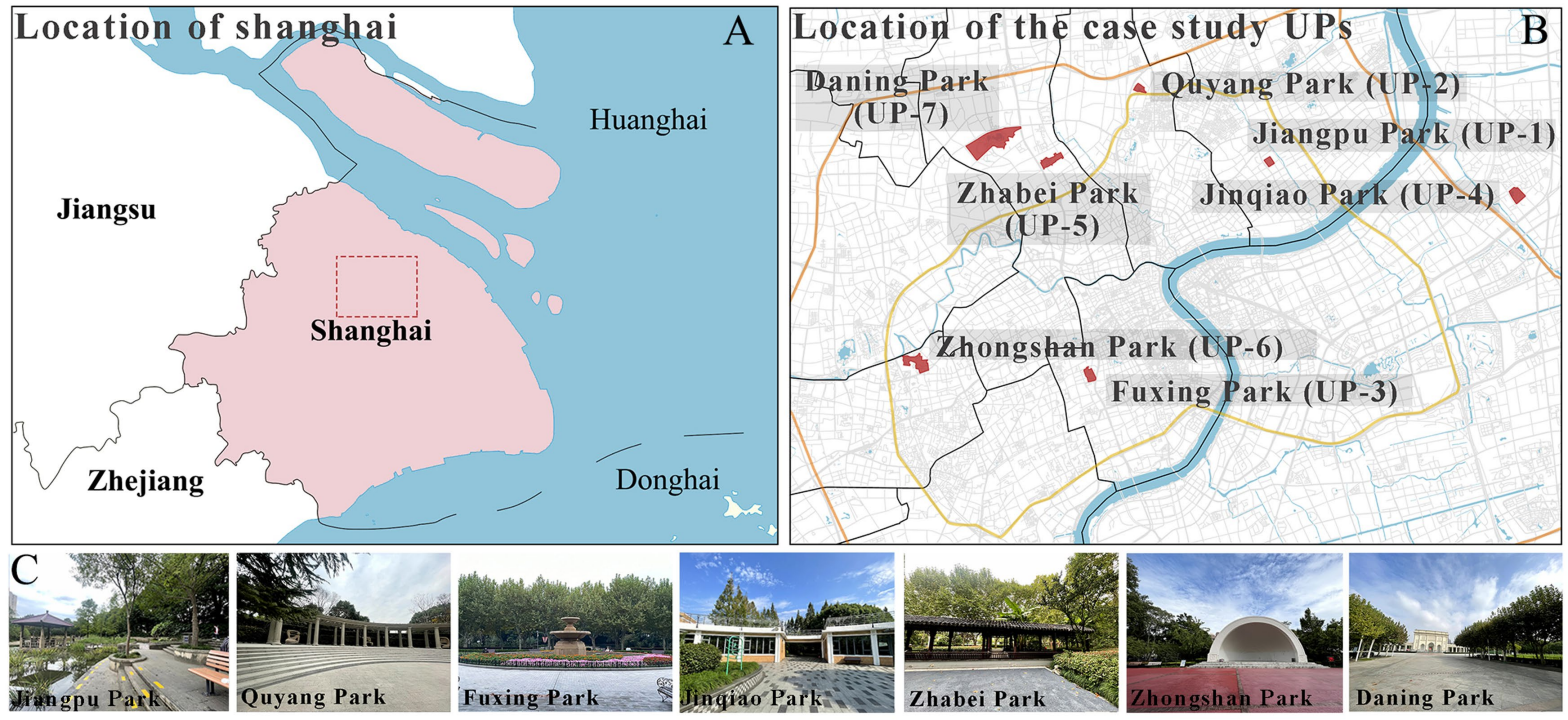
Case study city of Shanghai and selected urban parks. **(A)** Location of Shanghai, **(B)** location of the case study Ups, **(C)** field photographs of the Ups.

In this study, four comprehensive parks and three community parks in Shanghai were selected as research sites (Figure 1). The selection criteria were as follows: (1) Parks located within the outer loop of the central urban area, with high accessibility (entrance within 500 meters of a subway station) and open access (free 24/7 access), ensuring uniform public availability across all sites; (2) Parks with high popularity (from the Shanghai Greening and City Appearance Administration’s online voting rankings), and annual visitor numbers exceeding 1 million, ensuring consistent foot traffic and a diverse user demographic; (3) Parks with a variety of functions, ensuring a wide range of facilities and spaces that support a variety of resident activities. Measurement spots were determined using these criteria: (1) The size of each measurement spot was determined to be a 30 m × 30 m square (approximately 0.1 hectare). This selection is justified on both theoretical and practical grounds. Theoretically, it adheres to the 25–30 m critical distance for human environmental perception established by Gehl (61) and Ashihara’s (62), and corroborated by its application as a standard design module in landscape architecture (62, 63). Practically, it provides an optimal scale for spatial analysis by offering high resolution without segmenting natural behavioral patterns. (2) Measurement spots are evenly distributed across the park, with the number determined by the park’s size; (3) Measurement spots are accessible and next to internal park roads; (4) Measurement spots are not at park entrances/exits; (5) Each measurement spot is at least a 3-min walk from others. Consequently, five measurement spots were chosen for each of Jiangpu, Quyang, and Fuxing Parks; 10 for Jinqiao Park; 12 for Zhabei Park; and 15 and 16 plots, respectively, for Zhongshan and Daning Parks (Table 1).

**Table 1:** General descriptions of research sites and selected measurement spots.

Site	Size (ha)	Annual visits (per capita)	Type	Measurement spots
Jiangpu Park (UP-1)	3.08	1,004,405	community park	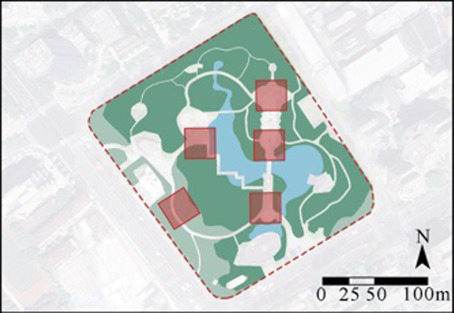
Quyang Park (UP-2)	6.47	1,613,330	community park	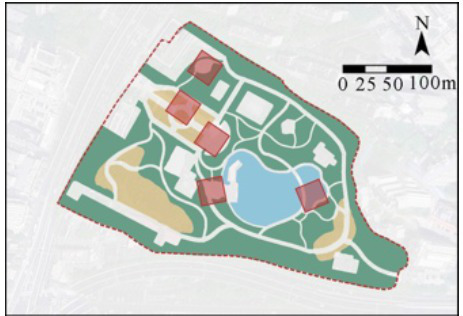
Fuxing Park (UP-3)	7.68	2,138,880	comprehensive park	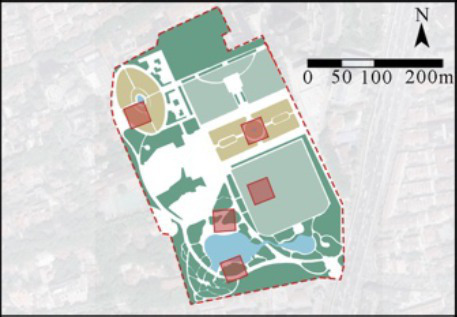
Jinqiao Park (UP-4)	11	2,814,500	community park	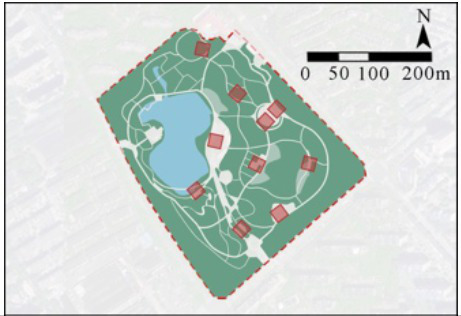
Zhabei Park (UP-5)	13.35	3,422,765	comprehensive park	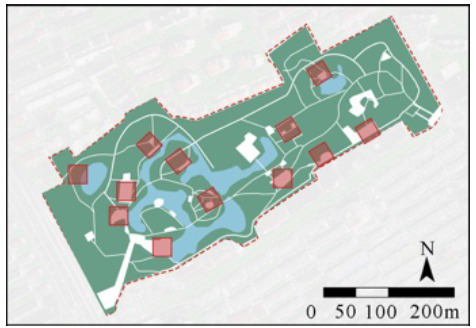
Zhongshan Park (UP-6)	20	2,814,500	comprehensive park	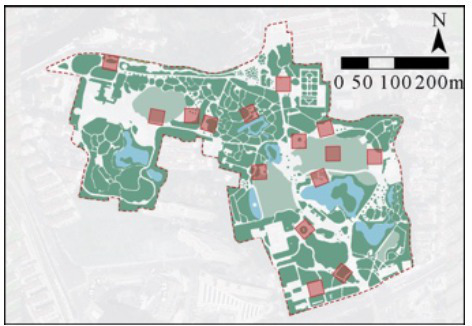
Daning Park (UP-7)	58.46	3,968,414	comprehensive park	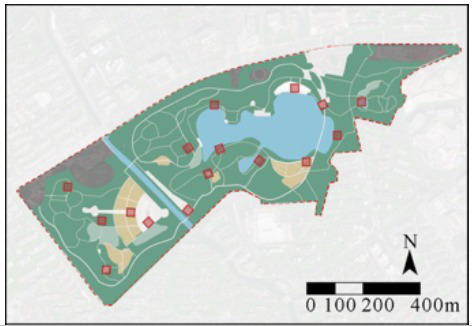

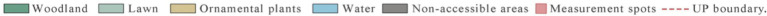

### 3.2 Data collection

#### 3.2.1 Landscape characteristics of measurement spots in urban parks

Upon finalizing the measurement plots, landscape characteristics were quantified through a combination of field photography and image semantic segmentation. Guided by the specific context of the study parks, China’s “Park Design Standards (GB 51221–2016),” and relevant literature (39, 64, 65), we identified 11 landscape features for analysis. These comprise both natural and artificial elements. Natural elements (sky, water, trees, shrubs, grass, and rough or uneven ground), which represent core biophilic components known to influence health behavior patterns (64). Artificial elements (pavement, buildings, resting facilities, service facilities, and shading facilities), which constitute key physical infrastructure and provide environmental affordances that support health-promoting activities and shape visitor experience (55, 56). Among these, “rough or uneven ground” was defined as the proportion of a plot covered by non-paved, minimally modified ground, such as bare soil, sparse vegetation, or gentle slopes. This variable was confirmed to be associated with park-based activities (66, 67).

**Figure 2:**
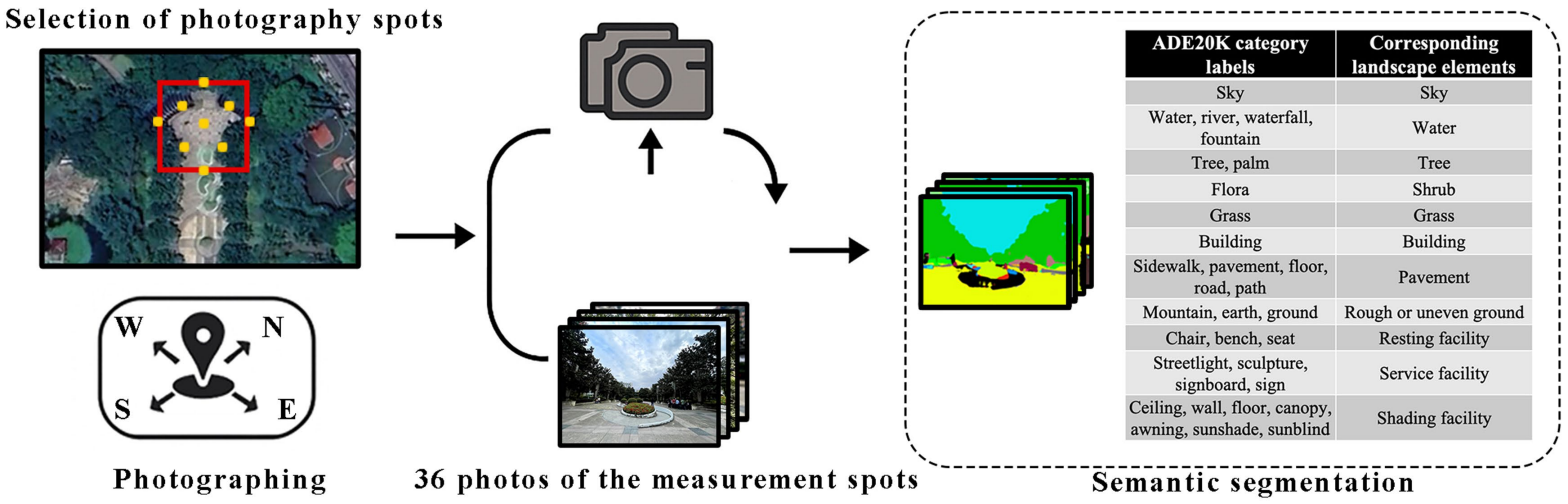
An illustration showing steps for photographing and semantic segmentation.

The on-site image collection protocol was adapted from the method proposed by Yin et al. (68), with a minimum of 9 shooting points selected within each measurement spot. At each point, photos were taken in four directions: east, south, west, and north. The photos had a 16:9 aspect ratio and a pixel size of 1920 × 1,080. Each measurement spot contained at least 36 photos. In total, 2,448 photos were obtained, meeting the image quantity requirements for semantic segmentation (69). These photos were then analyzed with semantic segmentation. Semantic segmentation is now widely used for analyzing images of urban environments like UP, open spaces, and streets (70–72), with datasets such as ADE20K (73) and Cityscapes (74) available. The image coding was conducted in a sequential procedure: (1) All 2,448 photos were analyzed using a Fully Convolutional Network (FCN) model, which segmented the images and assigned every pixel to one of the original ADE20K categories. Compared to the Cityscapes dataset, ADE20K includes more elements crucial to UPs (e.g., water bodies, vegetation, roads, buildings, and park facilities), making it better suited for studying how landscape elements and facilities impact activities within park settings (75). (2) The model’s initial output was then remapped into a simplified set of 11 landscape features relevant to our study (e.g., ‘tree’, ‘pavement’, ‘water’). (3) A final quantitative landscape profile was generated for each plot by calculating the average pixel percentages (Figure 2).

#### 3.2.2 Health behavior observed within measurement spots in urban parks

Research on human activities in public open spaces typically employs two methodological approaches. The first utilizes self-reported data from target populations (76) and questionnaires (77) to derive subjective insights. The second generates objective outcomes through researcher-conducted onsite observations, analysis of camera footage, and crowdsourced data. Direct onsite observation is particularly valuable for capturing granular behavioral and positional data, a critical requirement for this study’s focus on landscape features that support health-promoting behaviors. Following established methodologies (40, 51), this research adopts the System for Observing Play and Recreation in Communities (SOPARC) to record healthy activities within designated measurement areas. This validated tool demonstrates high efficacy in documenting spontaneous activity patterns in public spaces (78).

Aligned with the mental, physical, and social dimensions of health, healthy behaviors are categorized into three typologies (79): exercise, leisure, and social behaviors (summarized in Table 2). Trained observers conducted SOPARC-compliant data collection across selected sites over 2 months (October–November 2024). All observers received comprehensive training in the SOPARC protocol, covering the classification of health behaviors—namely exercise, leisure, and social activities—as well as techniques to minimize observer bias. Prior to formal data collection, they also completed pilot tests to ensure consistent application of behavioral coding criteria. Both the pre-field training and field implementation were standardized, incorporating synchronized observation procedures and the use of uniform mapping tools and recording forms. This approach effectively reduced inter-observer variability and ensured high procedural consistency across all study sites and time periods. Each park was observed on two sunny days (one weekday, one weekend), with three daily observation sessions: 8:00–10:00, 12:00–14:00, and 15:00–17:00. The observation timeslots were informed by the findings from the pilot study. Observations at each measurement point lasted approximately 15 min. The original SOPARC recording form is provided in Supplementary Table S1. Data on all observers, gender, age group, and health behavior category across three parks (UP-1, UP-2, UP-3) were collected to assess observational reliability by calculating the intraclass correlation coefficient (ICC) and interobserver agreement—a standard reliability monitoring approach used in SOPARC (78). The overall ICC for total users was 0.979, with 93.1% agreement among observers, reflecting a high level of consistency in observer performance. Furthermore, reliability metrics for gender, age, and behavioral category all exceeded the conventional threshold of >80% average percentage agreement, confirming the robustness of the observational measurements.

**Table 2:** Classification of health behaviors and corresponding codes.

Category	Sub-category (code)
Exercise behavior	Stretching (101), Tai Chi (102), bicycling (103), aerobic dance (104), badminton (105), running (106), frisbee playing (107), tennis (108), kickball (109), calisthenics (110), Tai Chi Chuan (111), social dancing (112), kicking shuttlecock (113), rope jumping (114), skating (115), scooter riding (116)
Leisure behavior	Sleeping (201), sitting (202), standing (203), drawing (204), walking (205), photography (206), chess game (207), card game (208), feeding animals (209), bird watching (210), playing with sand (211)
Social behavior	Reunion activities (301), group conversation (302), playing musical instruments (303), singing (304), parent–child activities (305), camping (306), children’s games (307)

### 3.3 Data analysis

Referring to the method proposed by Cohen et al. (43) for evaluating parks’ role in enhancing health and well-being, this study quantified health behavior intensity in each measurement spot using MET values for individual physical activities. MET values from the Adult Physical Activity Guidelines (80) were applied to calculate the recorded health behaviors. For activities not explicitly listed in the Adult Physical Activity Guidelines, such as scooter riding and shuttlecock kicking, the corresponding MET values were derived from previous relevant studies (81, 82). MET value used for each health behavior is listed in Supplementary Table S2. The overall ( OBI ), exercise ( EBI ), leisure ( LBI ), and social health behavior ( SBI ) intensity for each measurement spot were then calculated as follows (Equations 1–4).


$$\mathrm{OBI}=\sum_{i=1}^{n}({\mathrm{MET}}_{\mathrm{OB},i}\times N_{\mathrm{OB},i})$$ (1)

$$\mathrm{EBI}=\sum_{i=1}^{n}({\mathrm{MET}}_{\mathrm{EB},i}\times N_{\mathrm{EB},i})$$ (2)

$$\mathrm{LBI}=\sum_{i=1}^{n}({\mathrm{MET}}_{\mathrm{LB},i}\times N_{\mathrm{LB},i})$$ (3)

$$\mathrm{SBI}=\sum_{i=1}^{n}({\mathrm{MET}}_{\mathrm{SB},i}\times N_{\mathrm{SB},i})$$ (4)

Where $\;\mathrm{ME}T_{x,i}$ represents the METs value for the $i^{\mathrm{th}}$ activity in the respective behavior category x∈OB,EB,LB,SB . $N_{x,i}$ is the number of participants involved in the activity for behavior category x . The total number of activities in each behavior category is denoted by n .

To explore the relationship between landscape characteristics and health behavior intensity, the study employed multiple linear regression models. Four models were built using Python’s statsmodels, with exercise behavior intensity, leisure behavior intensity, social behavior intensity, and overall health behavior intensity as dependent variables, and landscape features as independent variables. Prior to regression analysis, Spearman correlation analysis was conducted to examine the relationships between continuous variables and dependent variables. To ensure the reliability of the regression models, multicollinearity among independent variables was assessed. Variables with a Variance Inflation Factor (VIF) below 10 and relevant correlations were included in the model construction.

**Figure 3:**
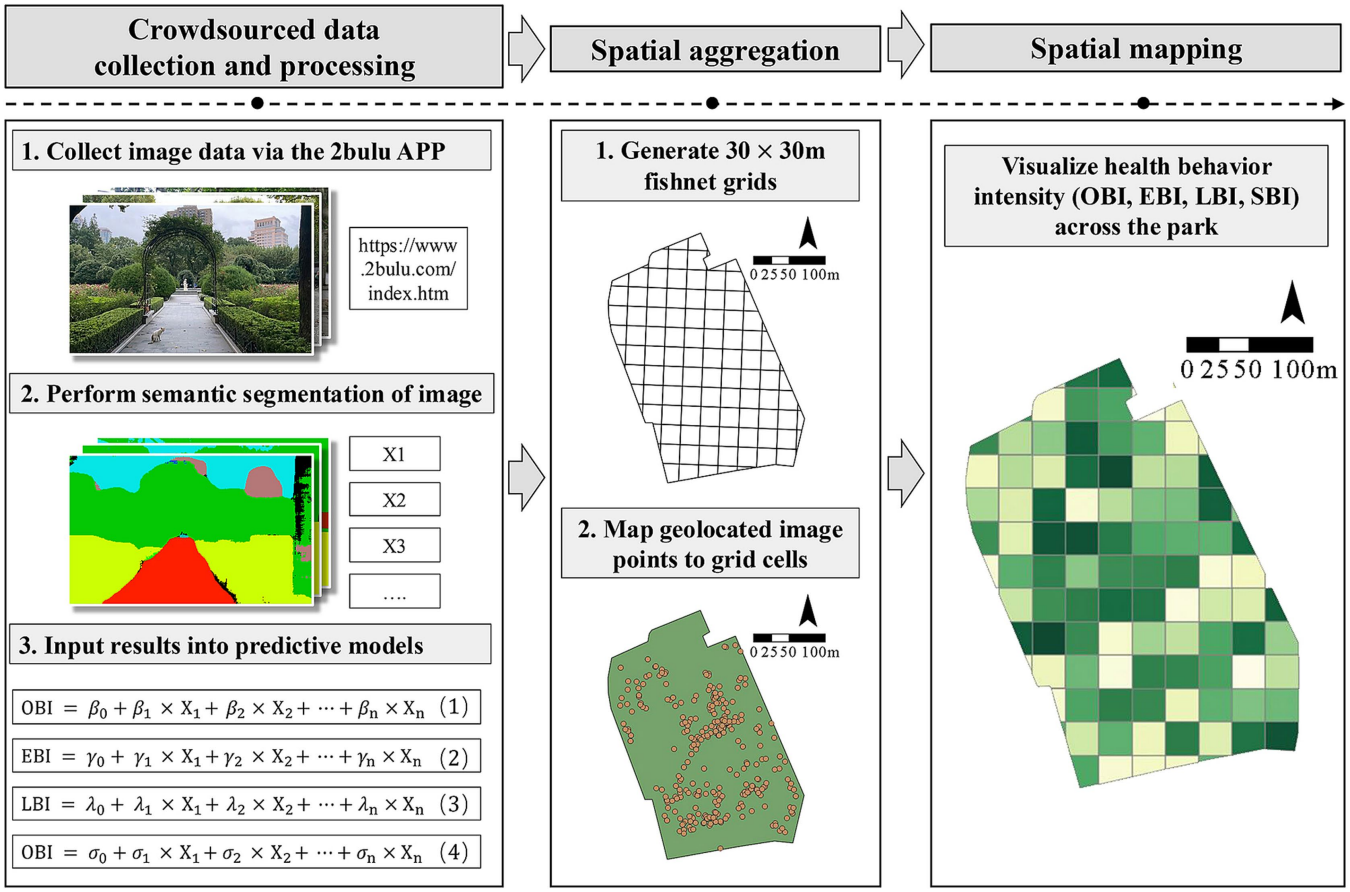
The process of predicting and mapping health behavior in Fuxing Park.

The model was then applied to conduct a full scope spatial mapping of health behavior intensity in Fuxing Park, one of the research sites. First, crowdsourced image data was obtained via the 2bulu APP, a top-rated Chinese outdoor activity APP where users can upload geo-tagged photos and track movements, to measure its landscape characteristics. After filtering out low resolution (dpi < 1080p), duplicate, and non-landscape-related images, a total of 467 images were retained for further analysis. Subsequently, a 30 m × 30 m fishnet analysis framework covering the study area was constructed in the ArcGIS Pro environment. Through spatial joining, the geo-tagged image points were precisely assigned to their corresponding grid cells. For grid cells containing multiple images, the landscape element values from all internal images were extracted and averaged to serve as the representative landscape feature for that cell. These averaged feature values were then input into the four predictive models established during the previous phase of the study to calculate corresponding OBI, EBI, LBI and SBI scores for each grid cell. Finally, to visualize the spatial distribution of health behavior intensity across Fuxing Park, the Natural Breaks (Jenks) method was employed to classify the scores of all grid cells, and spatial distribution maps of health behavior intensity were generated (Figure 3).

## 4 Results

### 4.1 Descriptive analysis

#### 4.1.1 The characteristics of landscape environment in research sites

Overall, the study area was characterized by a predominantly natural landscape. Vegetation constituted the primary component, with trees representing the most prevalent visual element (M = 36.26%), followed by shrubs (M = 6.05%) and grass (M = 4.59%). Among hardscape features, pavement accounted for a substantial proportion (M = 12.89%). In contrast, elements such as water bodies and built facilities (e.g., for resting and services) were minimally represented, each comprising less than 2.5% of the total area. A detailed statistical summary of all landscape components is provided in Table 3.

**Table 3:** Descriptive analysis for landscape characteristics in case study parks.

Landscape elements		Mean	UP-1	UP-2	UP-3	UP-4	UP-5	UP-6	UP-7
Sky	Mean	14.76%	15.55%	17.82%	6.07%	12.38%	9.33%	14.38%	22.20%
	Std. Dev	0.09	0.10	0.06	0.08	0.04	0.05	0.09	0.09
Water	Mean	2.07%	1.62%	1.64%	3.64%	0.82%	2.08%	0.88%	3.74%
	Std. Dev	0.03	0.01	0.03	0.03	0.01	0.03	0.01	0.05
Tree	Mean	36.26%	31.27%	26.94%	41.51%	44.65%	37.19%	39.11%	30.36%
	Std. Dev	0.11	0.09	0.07	0.08	0.06	0.07	0.10	0.13
Shrub	Mean	6.05%	8.80%	7.16%	8.83%	9.35%	7.48%	3.66%	3.08%
	Std. Dev	0.04	0.03	0.03	0.08	0.03	0.02	0.03	0.03
Grass	Mean	4.59%	3.98%	1.01%	6.87%	3.23%	3.30%	5.43%	6.23%
	Std. Dev	0.06	0.06	0.01	0.09	0.03	0.02	0.08	0.05
Building	Mean	2.88%	2.41%	4.64%	5.05%	1.02%	3.97%	3.01%	2.00%
	Std. Dev	0.02	0.01	0.02	0.03	0.01	0.03	0.02	0.02
Pavement	Mean	12.89%	14.51%	14.77%	7.76%	11.63%	14.58%	15.95%	10.03%
	Std. Dev	0.07	0.06	0.07	0.03	0.05	0.08	0.08	0.05
Rough or uneven ground	Mean	5.48%	2.99%	5.86%	4.92%	5.57%	6.02%	4.72%	6.55%
	Std. Dev	0.04	0.01	0.02	0.05	0.02	0.04	0.04	0.05
Resting facility	Mean	0.14%	0.11%	0.04%	0.26%	0.08%	0.10%	0.19%	0.15%
	Std. Dev	0.00	0.00	0.00	0.00	0.00	0.00	0.00	0.00
Service facility	Mean	0.05%	0.06%	0.09%	0.09%	0.03%	0.07%	0.04%	0.04%
	Std. Dev	0.00	0.00	0.00	0.00	0.00	0.00	0.00	0.00
Shading facility	Mean	4.31%	4.77%	4.06%	4.05%	2.51%	5.35%	4.15%	4.34%
	Std. Dev	0.05	0.05	0.03	0.05	0.01	0.07	0.03	0.04

Analysis of landscape composition revealed a clear spectrum among the seven urban parks. At one extreme, parks with high naturalness (UP-3, UP-4) were dominated by dense vegetation, featuring combined tree and shrub coverage exceeding 50%. The opposite extreme comprised parks with a distinctly open and built character (UP-1, UP-2, UP-7), which had significantly higher proportions of pavement and minimal tree cover. Occupying an intermediate position, UP-5 and UP-6 demonstrated a hybrid configuration of natural and artificial features. Detailed quantitative data supporting this classification are presented in Table 3.

#### 4.1.2 The characteristics of health behaviors observed in research sites

**Figure 4:**
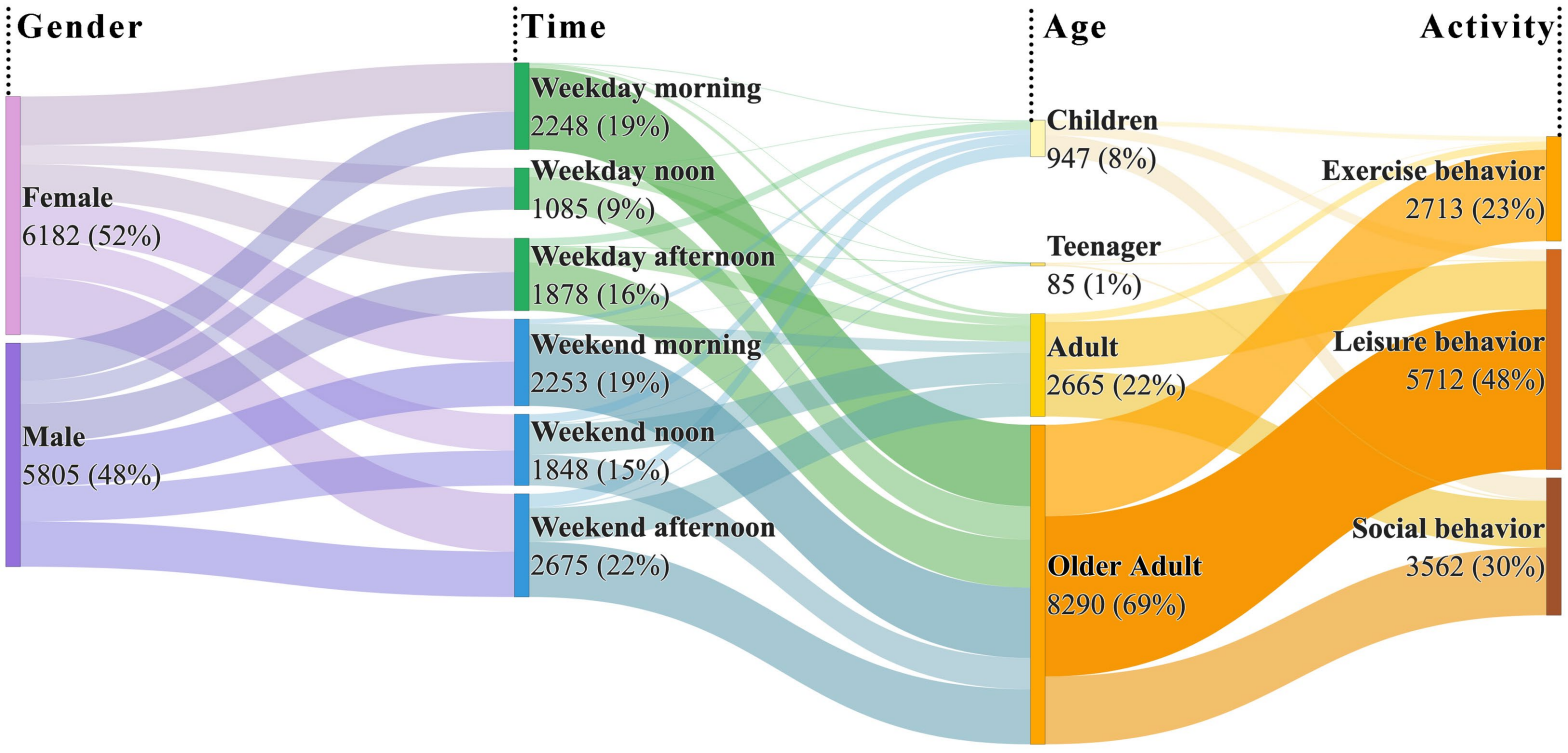
Characteristics of health behaviors observed in research sites.

During the observation period, 11,987 visitors were recorded. Older adults were the primary users, constituting 69% of all visitors, followed by adults at 22%. Children and adolescents accounted for only 8 and 1%, respectively. Regarding gender, female visitors slightly outnumbered males (52% vs. 48%), with this disparity being most pronounced among adults. Analysis of visitation patterns revealed a clear temporal distribution, with significantly higher park usage on weekends (56%) compared to weekdays (44%). Attendance peaked during morning and evening hours across all days. A demographic segmentation showed distinct temporal preferences: older adults concentrated their visits in early mornings consistently throughout the week, while adults, adolescents, and children primarily utilized the parks during weekend afternoons. Additionally, substantial attendance was observed among children (21%) and adults (16%) during weekday afternoons, as detailed in Figure 4.

**Figure 5:**
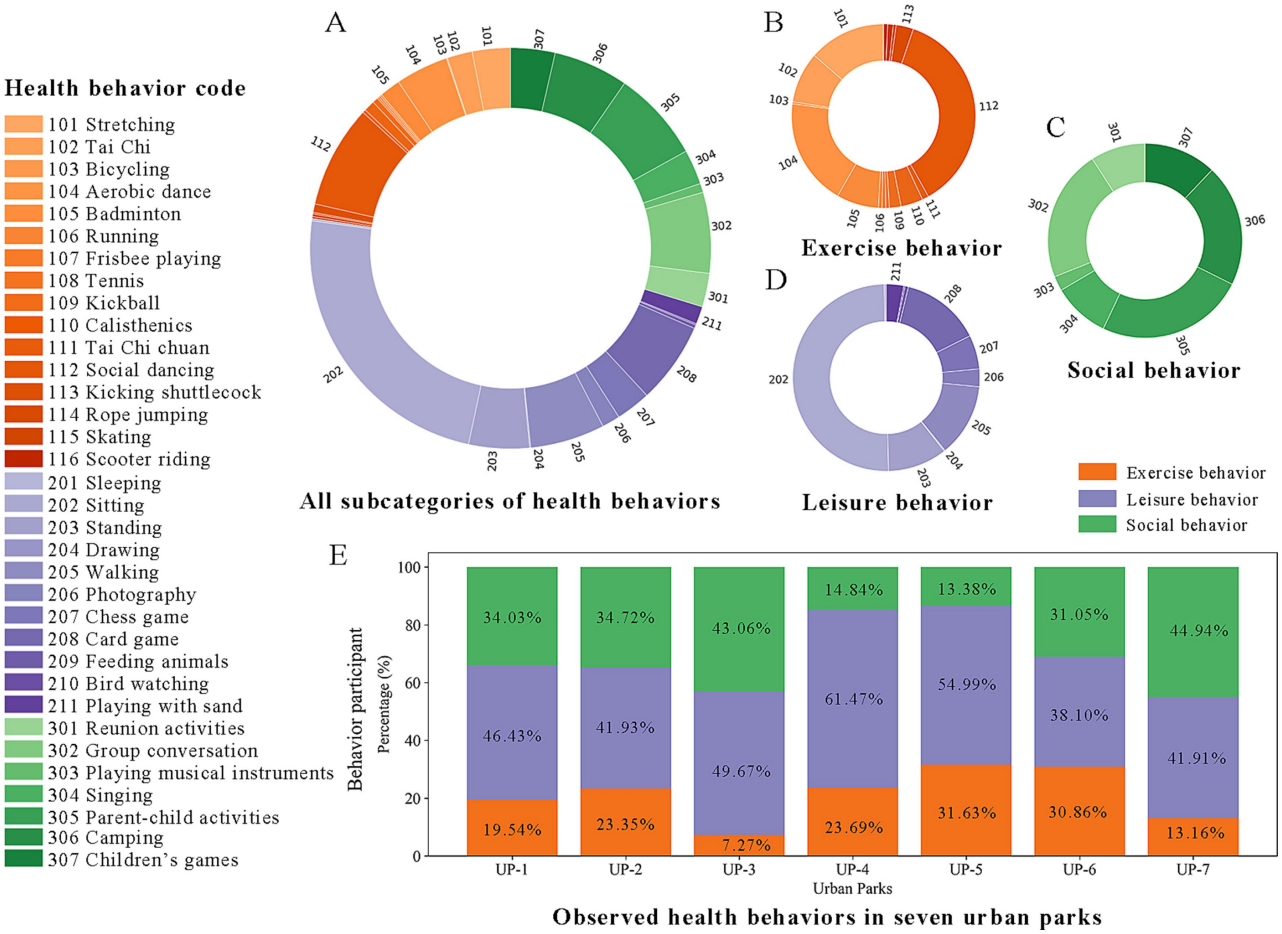
The characteristics of recorded health behaviors. **(A)** All categories of health behaviors, **(B)** exercise behaviors, **(C)** social behaviors, **(D)** leisure behaviors, **(E)** observed health behaviors in seven urban parks.

The study documented 34 subcategories of health behaviors. Leisure behaviors were most common (48%), with resting (*n* = 2,857; 23.83%), card games (*n* = 788; 6.57%), and walking (*n* = 721; 6.01%) being the most frequent (Figures 5A,D). Social behaviors ranked second (30%), with parent–child activities (*n* = 879; 7.33%), group conversations (*n* = 781; 6.52%), and camping (*n* = 724; 6.04%) being the top three categories (Figures 5A,C). Exercise behaviors were highlighted by social dancing (*n* = 995; 8.30%), aerobics (*n* = 510; 4.25%), and stretching (*n* = 368; 3.07%; Figures 5A,B; Supplementary Table S3).

Crucially, these general behavioral patterns were significantly shaped by the distinct landscape profiles of the parks. A clear association emerged between park naturalness and the prevalence of leisure activities, which were most common in highly natural settings such as UP-4 and relatively less frequent in more built-up environments like UP-2 and UP-7. Exercise behaviors, particularly structured activities such as aerobic and ballroom dancing, were most prominent in parks with a balanced mix of green spaces and built infrastructure (e.g., UP-5, UP-6). Furthermore, the nature of social behaviors varied with landscape context: vegetation-rich parks (e.g., UP-3) attracted reunion activities, while expansive open parks (e.g., UP-7) were predominantly used for camping. Together, these findings illustrate how specific landscape characteristics promote and diversify health-related behavioral patterns (Figure 5E).

#### 4.1.3 The intensity of health behaviors in research sites

Health behavior intensity for each observation spot was calculated based on behavior frequency and corresponding MET values. Overall, Exercise Behavior Intensity (EBI) was the highest (M = 192.91), followed by Social Behavior Intensity (SBI; M = 145.55), while Leisure Behavior Intensity (LBI) registered the lowest values (M = 128.47). Considerable variation in Overall Behavior Intensity (OBI) was also observed across parks (SD = 253.36), as summarized in Table 4.

**Table 4:** Descriptive statistics for dependent variables.

Dependent variables	Statistics	Overall	UP-1	UP-2	UP-3	UP-4	UP-5	UP-6	UP-7
OBI	Mean	463.98	523.30	487.34	460.58	470.86	482.25	465.49	419.79
	SD	253.36	253.17	175.55	315.84	208.80	222.33	311.46	279.50
EBI	Mean	192.91	148.30	189.80	92.02	228.14	263.03	252.38	108.98
	SD	187.37	151.38	134.28	58.66	193.84	184.42	239.99	154.47
LBI	Mean	128.47	182.30	119.94	177.60	172.47	137.83	80.27	109.62
	SD	84.11	45.51	95.01	88.52	124.12	65.18	49.04	78.02
SBI	Mean	145.55	192.70	177.60	190.96	70.25	81.39	132.84	213.70
	SD	170.37	91.36	85.15	241.85	49.77	60.95	191.09	240.07

The analysis of health behavior intensity across the urban parks further underscored how distinct environmental contexts differentially influence park usage. Consistent with participation data, parks featuring a balanced integration of green and built elements (e.g., UP-5, UP-6) not only attracted the largest number of individuals for exercise but also recorded the highest Exercise Behavior Intensity (EBI) scores, underscoring their dual capacity to promote both participation frequency and intensity in physical activities. A notable divergence emerged in Leisure Behavior Intensity (LBI): despite having the highest leisure participation, UP-4 did not yield the highest LBI—a result potentially attributable to its dense vegetation encouraging more sedentary, low-MET activities such as sitting. The pattern for Social Behavior Intensity (SBI) was more complex, with elevated values observed in both expansive open parks (e.g., UP-1, UP-7) and densely vegetated naturalistic settings (e.g., UP-3), suggesting that high-intensity social engagement can be supported by diverse landscape typologies.

### 4.2 Predicting health behavior intensity with landscape characteristics

Spearman correlation analysis was first used to investigate the relationship between landscape features of the research site and health behavior intensity. The results demonstrated that OBI was significantly positively correlated with ‘sky’ and ‘pavement’, and negatively correlated with ‘rough or uneven ground’ and ‘water’. EBI exhibited significant positive correlations with ‘tree’, ‘pavement’, and ‘resting facility’, and negative correlations with ‘water’, ‘grass’, and ‘rough or uneven ground’. LBI had fewer significant correlations, being positively correlated with ‘shrub’ and negatively correlated with ‘rough or uneven ground’. SBI displayed significant positive correlations with ‘sky’, ‘building’, and ‘service facility’, and negative correlations with ‘tree’, ‘shrub’, and ‘rough or uneven ground’. Among all the landscape elements, ‘rough or uneven ground’ was the only landscape feature that exhibited negative correlations with all four health behavior indicators. In contrast, ‘shading facility’ was not significantly correlated with health behavior (Table 5).

**Table 5:** Correlation between health behavior intensity and landscape characteristics of urban parks.

Landscape elements	OBI		EBI		LBI		SBI	
	Pearson’s r	*p*	Pearson’s r	*p*	Pearson’s r	*p*	Pearson’s r	*p*
Sky	0.26*	0.04	−0.15	0.23	0.018	0.88	0.59**	0.00
Water	−0.24*	0.05	−0.49**	0.00	0.15	0.22	−0.04	0.78
Tree	−0.18	0.14	0.24*	0.05	−0.15	0.21	−0.56**	0.00
Shrub	−0.19	0.13	0.01	0.93	0.25*	0.04	−0.31*	0.01
Grass	−0.10	0.42	−0.32**	0.01	0.04	0.75	0.14	0.26
Building	0.22	0.07	0.15	0.23	0.07	0.58	0.26*	0.03
Pavement	0.32**	0.01	0.56**	0.00	−0.00	0.10	0.04	0.72
Rough or uneven ground	−0.43**	0.00	−0.32**	0.01	−0.25*	0.04	−0.32**	0.01
Resting facility	0.22	0.08	0.27*	0.03	0.11	0.36	0.06	0.66
Service facility	0.08	0.50	0.02	0.86	0.06	0.61	0.24*	0.05
Shading facility	0.11	0.36	0.08	0.51	−0.03	0.81	0.13	0.28

(1) r values indicate the strength and direction of the correlation. (2) *p* values assess the statistical significance of the relationship, with values less than 0.05 indicating a significant association. (3) indicator values with * and ** are those landscape elements with significant relationship with health behavior intensity.

To explore the relationships between landscape characteristics and the four types of health behavior intensities, four multiple linear regression models were developed based on landscape factors related to health behavior intensity (Table 6). All models showed low multicollinearity, with tolerances above 0.1 and VIF values below 10. The F-tests for all four models (*p* < 0.01) indicated significant linear explanatory power of at least one predictor variable for each outcome variable.

**Table 6:** Multiple linear regression models and the significant influences.

Dependent variables	Independent variables	Estimate B	Std. error	t-value	*p-*value
OBI (R^2^ = 0.297, adjusted R^2^ = 0.252) Prob (F-statistic): 0.000157	(Constant)	281.63	130.46	2.16	0.04
	Sky	857.13	331.32	2.59	0.01
	Water	812.55	973.18	0.84	0.41
	Pavement	1040.91	505.92	2.06	0.04
	Rough or uneven ground	−1736.40	873.98	1.99	0.05
EBI (R^2^ = 0.463, adjusted R^2^ = 0.410) Prob (F-statistic): 6.86e-07	(Constant)	−167.14	124.97	1.34	0.19
	Water	539.54	711.73	0.76	0.45
	Tree	762.37	206.27	3.70	0.00
	Grass	147.14	402.78	0.37	0.72
	Pavement	1462.50	400.58	3.65	0.00
	Rough or uneven ground	−1553.52	606.02	2.56	0.01
	Resting facility	−27547.01	12413.63	2.22	0.03
LBI (R^2^ = 0.134, adjusted R^2^ = 0.108) Prob(F-statistic): 0.00918	(Constant)	119.00	23.15	5.14	0.00
	Shrub	585.04	237.29	2.47	0.02
	Rough or uneven ground	−473.24	261.63	1.81	0.08
SBI (R^2^ = 0.433, adjusted R^2^ = 0.377) Prob (F-statistic): 3.24e-06	(Constant)	−106.58	210.36	0.51	0.61
	Sky	1261.75	389.71	3.24	0.00
	Tree	118.39	333.11	0.36	0.72
	Shrub	−540.11	452.20	1.19	0.24
	Building	1447.28	1163.77	1.24	0.22
	Rough or uneven ground	152.50	539.41	0.28	0.78
	Service facility	10657.09	36168.39	0.30	0.77

The regression model for Overall Behavior Intensity (OBI) accounted for 25.2% of the total variance (R^2^ = 0.252), revealing that behavioral intensity is primarily driven by a combination of visual openness and physical accessibility. Specifically, higher sky visibility and greater pavement coverage emerged as significant positive predictors, whereas rough or uneven ground exerted a negative influence, as detailed in Equation 5.


$$\begin{array}{l} \mathrm{OBI}=281.63+857.13\times \mathrm{Sky}+1040.91\\ \times \text{Pavement}-1736.40\times \text{Rough or uneven ground} \end{array}$$ (5)

The model for Exercise Behavior Intensity (EBI) accounted for a substantial 41.0% of the variance (R^2^ = 0.410), indicating that higher-intensity exercise is supported by environments combining accessible, unobstructed surfaces and visually pleasant greenery. This relationship is reflected in the significant positive association with ‘pavement’ and ‘trees’, while ‘rough or uneven ground’ and ‘resting facilities’ emerged as negative predictors, as specified in Equation 6.


$$\begin{array}{l} \mathrm{EBI}=-167.14+762.37\times \text{Tree}+1462.50\times \text{Pavement}-1553.52\\ \times \text{Rough or uneven ground}-27547.01\times \text{Resting facility} \end{array}$$ (6)

Of the landscape features analyzed, only shrub coverage demonstrated a significant association with Leisure Behavior Intensity (LBI), implying that the spatial enclosure and visual interest afforded by shrubs may promote more active forms of leisure engagement. A model was constructed accordingly with an *R*^2^ of 0.108 in Equation 7:


LBI=119.00+585.04×Shrub (7)

The analysis revealed that Social Behavior Intensity (SBI) demonstrated a significant association with sky visibility. This finding underscores the role of open, unobstructed spaces in facilitating social interactions. A linear model with an *R*^2^ of 0.377 appeared in Equation 8.


SBI=−106.58+1261.75×Sky (8)

### 4.3 Mapping the health behavior in Fuxing park

**Figure 6:**
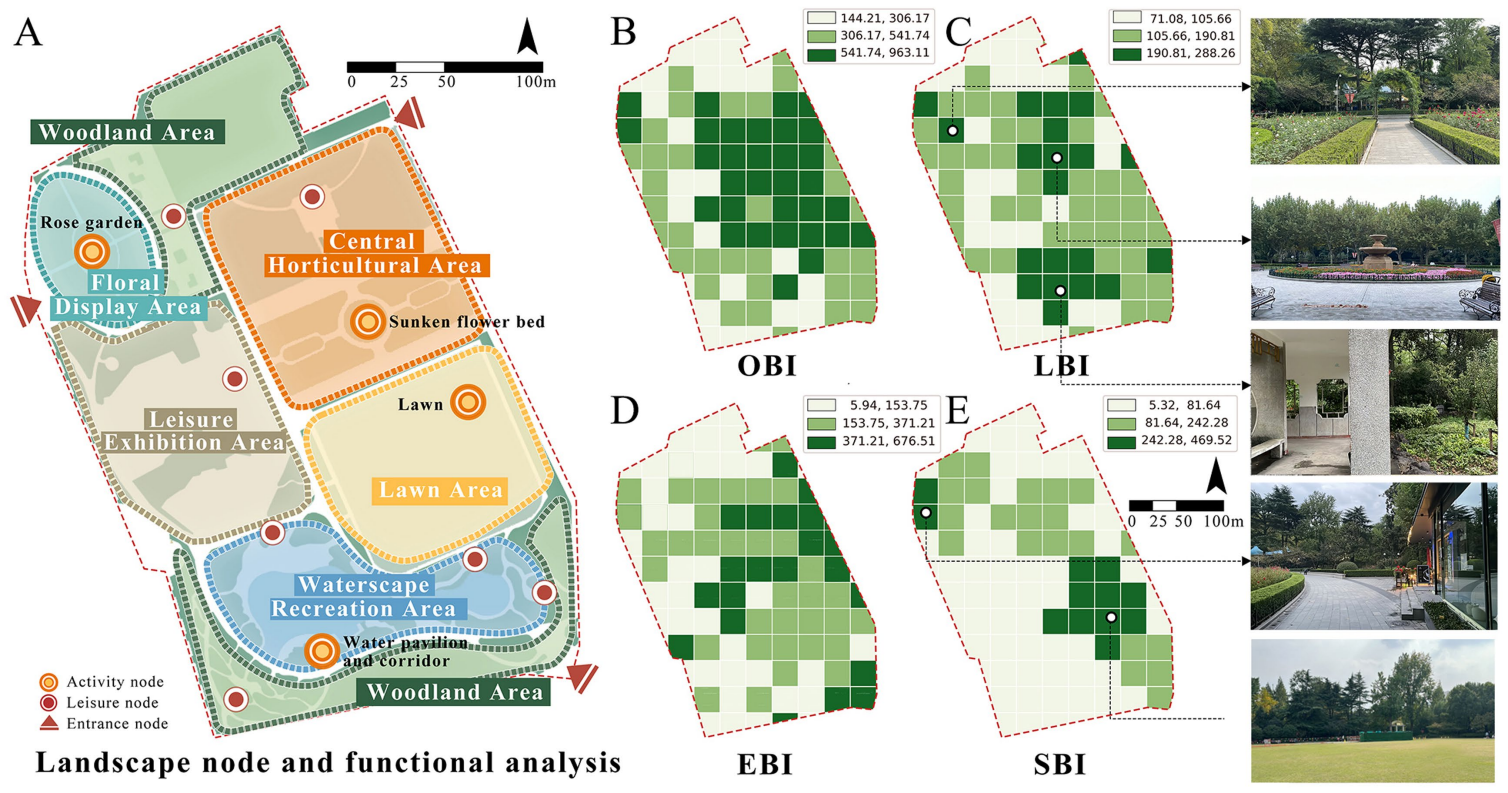
Spatial mapping of OBI, EBI, LBI, and SBI in Fuxing Park. **(A)** Landscape node and functional analysis, **(B)** OBI, **(C)** LBI, **(D)** EBI, **(E)** SBI.

Employing Fuxing Park as a demonstrative case, crowdsourced image-derived landscape metrics were processed through multivariate regression models to generate spatial distributions of four behavioral indices: OBI, EBI, LBI, and SBI (Figure 6). This computational mapping revealed significant intra-park stratification of health behavior hotspots, demonstrating the framework’s capacity to resolve microscale behavioral-environmental interactions.

Results reveal pronounced spatial stratification in terms of three types of health behaviors: OBI peaks in central-northern sectors characterized by expansive sky visibility, turfgrass matrices, and hardscape plazas; EBI concentrates centrally with secondary clusters in southeastern entrance plazas and arbor-lined eastern pathways; LBI intensifies in southern waterscapes and central horticultural zones featuring dense shrubbery and restorative seating clusters; SBI dominates southeastern lawns with spatial congruence between high-intensity zones and turf coverage (Figure 6).

Cross-behavior analysis demonstrates that functional polyvalence in central sectors where EBI-LBI overlap indicates multifunctional spaces supporting both exercise and leisure activities. Also, spatial exclusivity between EBI and SBI—vigorous exercise localizes in arboreal corridors while social gatherings occupy open lawns and shaded congregation nodes—revealing competitive partitioning driven by divergent environmental requirements. Besides, the behavioral compatibility of LBI exhibits neutral coexistence with both exercise and social activities, underscoring its adaptive spatial logic (Figure 6).

## 5 Discussion

Taking small-scale measurement spots as the fundamental units, this study constructs an analytical model to investigate the relationship between environmental landscape characteristics and user health behaviors. The aim is to reveal the differential impacts of landscape feature heterogeneity within urban parks on health behaviors. Leveraging technologies such as crowdsourced geospatial data and GIS analysis, the model calculates and visualizes the types and intensity of health behaviors across the entire park area using small-sample data. Thus, it provides a basis for health-related decision-making both for users seeking to enhance their health through parks and also, for designers, and managers aiming to improve the health benefits of park spaces.

### 5.1 A health-led approach for designing urban parks

A major finding of this study is the identification and comparison of how plot-level landscape features support distinct health behaviors within urban parks. Analysis revealed that leisure and social behaviors showed significant positive correlations with sky visibility, consistent with existing research indicating that open sky vistas enhance visual comfort, spatial perception, and align with preferences for stationary activities (60), thereby promoting social interactions. Exercise behavior intensity, however, was primarily associated with higher proportions of trees—areas providing shade, airflow, and favorable microclimates for activities like stretching and aerobics (83, 84). Conversely, shrubs demonstrated greater influence on leisure activities by fostering tranquility, creating semi-private spaces, and fulfilling the prospect-refuge dynamic essential for relaxation (85). Rough or uneven ground consistently hindered all three behavior types, confirming earlier findings that irregular ground textures restrict movement and reduce environmental preference (40, 67). Although uneven surfaces have been linked to greater tactile engagement in specific contexts like mountains or trails, these effects are largely inapplicable to general park-based behaviors (66).

This research also yielded findings contrasting with established literature, offering new perspectives on park design. Notably, no significant link was found between lawn area and social behavior intensity—a departure from prior studies (86). This discrepancy is potentially explained by practices in Shanghai parks (including the study site), where lawns are often restricted as visual-only zones, conditioning visitors to perceive them as non-activity spaces. Similarly, resting facilities correlated negatively with exercise behavior intensity, contradicting studies suggesting benches promote activity (87); plot-scale analysis indicates these areas prioritize sedentary or social uses, conflicting with fitness needs. Service facilities also showed no significant impact on social behaviors, challenging assumptions about their inherent social value and suggesting design quality outweighs mere presence (88, 89). These deviations underscore the context-dependent nature of environment-behavior relationships.

The case study of Fuxing Park empirically demonstrates that health behavior intensity maps provide effective, data-informed guidance for resource allocation, maintenance planning, and design renovations. At the management level, health behavior intensity maps directly inform these strategies: High-activity zones warrant prioritized maintenance and cleaning to ensure landscape and facility quality, while areas with high LBI values may require enhanced lighting and safety infrastructure to better support predominantly older users engaged in leisure activities. Furthermore, park managers can leverage map data to strategically schedule temporary programming—such as community events or fitness classes—in areas exhibiting high SBI. At the planning level, the maps facilitate the integration of different park zones, preventing spatial exclusivity between activities. For example, in central park areas in Fuxing Park, where social and exercise activities exhibit clear spatial exclusion, multifunctional spaces or buffer zones can be introduced to mitigate these conflicts. Moreover, during park renovation projects, planners can utilize the OBI maps as a tool for pre- and post-renovation evaluation, providing empirical evidence of the effectiveness of the changes. At the design level, the framework allows for targeted spatial optimization by modifying specific landscape features. Low-intensity areas can be revitalized by introducing elements that boost their attractiveness (e.g., adding paved pathways in low-OBI zones to promote health behaviors). Conversely, high-intensity zones can be enhanced to better support their dominant activities, such as installing high-quality service facilities (e.g., cafes or improved restrooms) in high-SBI areas to encourage social interaction.

Collectively, these findings validate the complex, dynamic interplay where human behavior and environmental features mutually adapt. They emphasize the critical need for designers and managers to prioritize localized contexts and nuanced design qualities—rather than applying universal prescriptions—to effectively foster diverse health behaviors in urban parks. Recognizing site-specific patterns (e.g., rough or uneven ground, culturally influenced lawn usage) and subtle feature interactions (e.g., spatial competition between facilities and activities) is essential for optimizing park health benefits.

### 5.2 A more detailed park prescription for urban residents

Findings at the park plot scale offer finer-grained guidance for Park Prescription Programs (Park Rx). First proposed in the US in 2009 (90), Park Rx aims to improve health and well-being by encouraging time spent in nature. However, prescriptions provided by healthcare professionals are often vague, typically specifying only duration, frequency, and generic exercise types, without addressing where specific activities should optimally occur within parks. This lack of spatial specificity can lead visitors to expend considerable effort locating suitable spaces. A growing body of research indicates that personalized prescriptions, tailored to both specific park environments and targeted health behaviors, yield greater health benefits (8, 91). Developing such precision prescriptions necessitates an in-depth understanding of the spatial distribution of health behaviors within parks. To address this need, our study leverages a standardized analytical framework at the plot scale, providing both data support and visualization tools essential for generating precise, health-behavior-oriented guidance.

This study integrates regression modeling with crowdsourced image data to quantify and predict spatial patterns of health behavior intensity across urban parks, demonstrating how heterogeneous landscapes support distinct categories of health activities. Building on these insights, clinicians and patients can collaboratively develop personalized park prescriptions specifying optimal locations, activity types, and engagement frequencies. For instance, evidence indicates that multimodal interventions combining physical activity (e.g., walking) with relaxation practices (e.g., meditation) enhance patient outcomes (91). Using our behavioral maps, a practitioner could guide patients to zones exhibiting overlapping Exercise and Leisure Behavior Intensity (EBI-LBI), thereby addressing individualized health needs. Additionally, by comparing intensity distributions across all three behavior types, users can identify potential environmental conflicts between activities to minimize experiential interference.

### 5.3 Research limits

Although the results largely met expectations and a plot-scale health behavior prediction model was successfully developed, the study has limitations in its experimental design and data analysis. Firstly, the research was conducted during a specific period (October–November), failing to cover all seasons or account for diurnal variations. Future research should extend the temporal scope to incorporate seasonal and diurnal influences, providing a more comprehensive reflection of park usage across the full cycle. Secondly, although the analysis followed an established, design-based sampling framework, the limited number of parks and plots studied means that some internal dependencies could not be completely ruled out. Furthermore, given that pocket parks and large suburban parks are recognized as significant venues for health behaviors (92, 93)—with usage patterns potentially different from the urban central parks targeted—future research should include more parks and employ multi-phase or longitudinal observations to expand upon this framework. Besides, while the model specification was aligned with objectives of this research, the cross-sectional and aggregated design cannot fully separate the effects of landscape attraction from user self-selection. Future studies could address this by incorporating individual-level tracking, longitudinal observations, or quasi-experimental approaches to better disentangle behavioral preferences from environmental influences. This would help formulate inclusive, context-responsive guidelines that account for demographic diversity (e.g., age and gender) in park planning.

Regarding data analysis, while the ADE20K dataset was used—covering major park landscape features—for semantic segmentation, it was not specifically trained on park environments. This may have resulted in insufficient recognition accuracy. Future work could benefit from developing a semantic segmentation training set dedicated to park scenes to improve feature quantification precision. Furthermore, the conducted analysis primarily focused on individual landscape elements, failing to comprehensively capture the combinatorial effects of landscape features or the influence of landscape quality attributes (such as visual depth, openness, and enclosure on health behaviors). Future research should further explore the interactive effects of landscape feature combinations.

## 6 Conclusion

Within park-based health interventions, plot-scale analysis of health behaviors is essential, yet existing research, predominantly focused on macro and meso scales and lacking standardized quantitative methods, imposes significant constraints on users’ activity location choices and managers’ efforts to enhance health benefits. Addressing this gap, this study developed a model utilizing plot-scale measurement and crowdsourced data to predict the spatial differentiation of health behaviors based on environmental characteristics and to generate health behavior intensity maps for one of the case study sites, Fuxing Park. The findings demonstrate that this plot-scale approach is instrumental in understanding the spatial patterning of diverse health behaviors within urban parks. Crucially, the resulting health behavior intensity maps provide vital insights for design and management strategies aimed at optimizing park health benefits. Collectively, this research delivers practical tools for advancing data-driven approaches to maximize the health-promoting potential of urban parks.

## Data Availability

The raw data supporting the conclusions of this article will be made available by the authors, without undue reservation.
