# Supplementary material for: A more detailed park prescription: How does landscape support human health behavior?
Source: Front Public Health. 2025 Dec 15;13:1696097. doi: 10.3389/fpubh.2025.1696097 (PMC12745453; doi:10.3389/fpubh.2025.1696097)
Supplement: Supplementary file 1 [file Data_Sheet_1.docx]

Supplementary Material

# Supplementary Table S1. The System for Observing Play and Recreation in Communities used for recoding health behavior.

| **Observation area information collection form** | | | | | | | | | |
| --- | --- | --- | --- | --- | --- | --- | --- | --- | --- |
| **UGS Name:** | **Sample plots ID:** | | | **Period:** | | | **Date:** | | |
| Category | | The number of participants in healthy behaviors | | | | | | | |
|  |  | Children | | Teenager | | Adult | | Senior | |
|  |  | Female | Male | Female | Male | Female | Male | Female | Male |
| Exercise behavior | Stretching |  |  |  |  |  |  |  |  |
|  | Tai chi |  |  |  |  |  |  |  |  |
|  | ... |  |  |  |  |  |  |  |  |
| Leisure behavior | Sleeping |  |  |  |  |  |  |  |  |
|  | Photography |  |  |  |  |  |  |  |  |
|  | ... |  |  |  |  |  |  |  |  |
| Social behavior | Camping |  |  |  |  |  |  |  |  |
|  | Children games |  |  |  |  |  |  |  |  |
|  | ... |  |  |  |  |  |  |  |  |

# Supplementary Table S2. The MET value adopted for each observed health behavior.

| **Category** | **Code** | **Health behavior** | **METs** | **Notes** | **References** |
| --- | --- | --- | --- | --- | --- |
| Exercise behavior | 101 | Stretching | 2.3 | mild | Herrmann et al., 2024 |
|  | 102 | Tai Chi | 3.3 | general |  |
|  | 103 | Bicycling | 7.0 | general |  |
|  | 104 | Aerobic dance | 4.8 | low impact, moderate effort |  |
|  | 105 | Badminton | 5.5 | social singles and doubles, general |  |
|  | 106 | Running | 7.8 | 4.3 to 4.8 mph |  |
|  | 107 | Frisbee playing | 3.0 | general |  |
|  | 108 | Tennis | 6.8 | general, moderate effort |  |
|  | 109 | Kickball | 7.0 |  |  |
|  | 110 | Calisthenics | 3.8 | e.g., push ups, sit ups, pull-ups, lunges), moderate effort |  |
|  | 111 | Tai Chi Chuan | 6.0 | Yang style |  |
|  | 112 | Social dancing | 5.5 | ballroom dancing, fast |  |
|  | 113 | Kicking shuttlecock | 5.0 | Kicking shuttlecock (Sepak Takraw) generally fall within the 4–5.5 MET range, depending on effort. | Udomtaku et al., 2020 |
|  | 114 | Rope jumping | 8.3 | slow pace | Herrmann et al., 2024 |
|  | 115 | Skating | 7.0 | rooler |  |
|  | 116 | Scooter riding | 5.7 | riding a mini scooter | Butte et al., 2017 |
| Leisure behavior | 201 | Sleeping | 1.0 |  | Herrmann et al., 2024 |
|  | 202 | Sitting | 1.0 | quietly, general |  |
|  | 203 | Standing | 1.3 | quietly |  |
|  | 204 | Drawing | 1.8 | drawing, writing, painting, standing |  |
|  | 205 | Walking | 3.5 | walking for pleasure |  |
|  | 206 | Photography | 2.5 | Photography, sitting or standing, corresponds to sedentary or light activity with an energy cost of 2–3 METs. |  |
|  | 207 | Chess game | 1.5 | chess game, watching chess play, sitting or standing |  |
|  | 208 | Card game | 1.5 | card game, watching card play, sitting or standing |  |
|  | 209 | Feeding animals | 2.5 |  |  |
|  | 210 | Bird watching | 2.5 | slow walk |  |
|  | 211 | Playing with sand | 1.5 | playing with sand, toys, water | Butte et al., 2017 |
| Social behaviors  · | 301 | Reunion activities | 1.5 |  | Herrmann et al., 2024 |
|  | 302 | Group conversation | 1.3 | talking in person, sitting or standing |  |
|  | 303 | Playing musical instruments | 2.0 | general |  |
|  | 304 | Singing | 2.0 |  |  |
|  | 305 | Parent-child activities | 3.5 | walk/run play with children, moderate, only active periods |  |
|  | 306 | Camping | 2.5 | involving standing, walking, sitting, light-to-moderate effort |  |
|  | 307 | Children’s games | 5.8 | moderate effort | Butte et al., 2017 |

**Reference**

[1] Herrmann SD, Willis EA, Ainsworth BE, Barreira TV, Hastert M, Kracht CL, et al. 2024 Adult Compendium of Physical Activities: A third update of the energy costs of human activities. Journal of Sport and Health Science 2024;13:6–12.

[2] Udomtaku K, Konharn K. Energy expenditure and movement activity analysis of sepaktakraw players in the Thailand league. Journal of Exercise Science & Fitness 2020;18:136–41.

[3] Butte NF, Watson KB, Ridley K, Zakeri IF, Mcmurray RG, Pfeiffer KA, et al. A Youth Compendium of Physical Activities: Activity Codes and Metabolic Intensities. Medicine and Science in Sports and Exercise 2017;50:246.

# Supplementary Table S3. Observed health behavior in seven UPs

| **Code** | **UP-1** | | **UP-2** | | **UP-3** | | **UP-4** | | **UP-5** | | **UP-6** | | **UP-7** | | **Total** | |
| --- | --- | --- | --- | --- | --- | --- | --- | --- | --- | --- | --- | --- | --- | --- | --- | --- |
|  | **N** | **%** | **N** | **%** | **N** | **%** | **N** | **%** | **N** | **%** | **N** | **%** | **N** | **%** | **N** | **%** |
| 101 | 13 | 1.37% | 22 | 2.69% | 7 | 0.67% | 77 | 3.87% | 84 | 4.01% | 101 | 3.91% | 64 | 2.55% | 368 | 3.07% |
| 102 | 24 | 2.52% | 15 | 1.83% | 9 | 0.86% | 41 | 2.06% | 67 | 3.20% | 64 | 2.48% | 23 | 0.92% | 243 | 2.03% |
| 103 | 7 | 0.74% | 0 | 0.00% | 1 | 0.10% | 1 | 0.05% | 0 | 0.00% | 1 | 0.04% | 0 | 0.00% | 10 | 0.08% |
| 104 | 0 | 0.00% | 0 | 0.00% | 12 | 1.15% | 15 | 0.75% | 195 | 9.32% | 151 | 5.85% | 137 | 5.46% | 510 | 4.25% |
| 105 | 4 | 0.42% | 18 | 2.20% | 2 | 0.19% | 53 | 2.67% | 51 | 2.44% | 42 | 1.63% | 31 | 1.24% | 201 | 1.68% |
| 106 | 5 | 0.53% | 10 | 1.22% | 0 | 0.00% | 0 | 0.00% | 0 | 0.00% | 1 | 0.04% | 1 | 0.04% | 17 | 0.14% |
| 107 | 1 | 0.11% | 0 | 0.00% | 1 | 0.10% | 0 | 0.00% | 0 | 0.00% | 16 | 0.62% | 0 | 0.00% | 18 | 0.15% |
| 108 | 0 | 0.00% | 1 | 0.12% | 0 | 0.00% | 0 | 0.00% | 2 | 0.10% | 5 | 0.19% | 9 | 0.36% | 17 | 0.14% |
| 109 | 6 | 0.63% | 6 | 0.73% | 13 | 1.24% | 1 | 0.05% | 0 | 0.00% | 22 | 0.85% | 7 | 0.28% | 55 | 0.46% |
| 110 | 0 | 0.00% | 74 | 9.05% | 0 | 0.00% | 2 | 0.10% | 30 | 1.43% | 0 | 0.00% | 0 | 0.00% | 106 | 0.88% |
| 111 | 0 | 0.00% | 43 | 5.26% | 0 | 0.00% | 14 | 0.70% | 4 | 0.19% | 6 | 0.23% | 7 | 0.28% | 74 | 0.62% |
| 112 | 119 | 12.50% | 2 | 0.24% | 30 | 2.87% | 253 | 12.73% | 198 | 9.46% | 336 | 13.01% | 16 | 0.64% | 954 | 7.96% |
| 113 | 0 | 0.00% | 0 | 0.00% | 0 | 0.00% | 11 | 0.55% | 29 | 1.39% | 33 | 1.28% | 10 | 0.40% | 83 | 0.69% |
| 114 | 3 | 0.32% | 0 | 0.00% | 0 | 0.00% | 0 | 0.00% | 2 | 0.10% | 2 | 0.08% | 5 | 0.20% | 12 | 0.10% |
| 115 | 4 | 0.42% | 0 | 0.00% | 1 | 0.10% | 3 | 0.15% | 0 | 0.00% | 14 | 0.54% | 4 | 0.16% | 26 | 0.22% |
| 116 | 0 | 0.00% | 0 | 0.00% | 0 | 0.00% | 0 | 0.00% | 0 | 0.00% | 3 | 0.12% | 16 | 0.64% | 19 | 0.16% |
| 201 | 3 | 0.32% | 0 | 0.00% | 6 | 0.57% | 1 | 0.05% | 1 | 0.05% | 4 | 0.15% | 6 | 0.24% | 21 | 0.18% |
| 202 | 129 | 13.55% | 120 | 14.67% | 206 | 19.71% | 308 | 15.49% | 769 | 36.74% | 663 | 25.67% | 662 | 26.40% | 2857 | 23.83% |
| 203 | 49 | 5.15% | 39 | 4.77% | 83 | 7.94% | 50 | 2.52% | 113 | 5.40% | 189 | 7.32% | 63 | 2.51% | 586 | 4.89% |
| 204 | 1 | 0.11% | 0 | 0.00% | 2 | 0.19% | 4 | 0.20% | 2 | 0.10% | 1 | 0.04% | 0 | 0.00% | 10 | 0.08% |
| 205 | 185 | 19.43% | 120 | 14.67% | 121 | 11.58% | 52 | 2.62% | 132 | 6.31% | 28 | 1.08% | 83 | 3.31% | 721 | 6.01% |
| 206 | 14 | 1.47% | 5 | 0.61% | 71 | 6.79% | 11 | 0.55% | 2 | 0.10% | 43 | 1.66% | 29 | 1.16% | 175 | 1.46% |
| 207 | 47 | 4.94% | 0 | 0.00% | 23 | 2.20% | 101 | 5.08% | 132 | 6.31% | 26 | 1.01% | 7 | 0.28% | 336 | 2.80% |
| 208 | 0 | 0.00% | 51 | 6.23% | 0 | 0.00% | 695 | 34.96% | 0 | 0.00% | 30 | 1.16% | 12 | 0.48% | 788 | 6.57% |
| 209 | 0 | 0.00% | 3 | 0.37% | 3 | 0.29% | 0 | 0.00% | 0 | 0.00% | 0 | 0.00% | 28 | 1.12% | 34 | 0.28% |
| 210 | 13 | 1.37% | 0 | 0.00% | 0 | 0.00% | 0 | 0.00% | 0 | 0.00% | 0 | 0.00% | 0 | 0.00% | 13 | 0.11% |
| 211 | 1 | 0.11% | 5 | 0.61% | 4 | 0.38% | 0 | 0.00% | 0 | 0.00% | 0 | 0.00% | 161 | 6.42% | 171 | 1.43% |
| 301 | 5 | 0.53% | 7 | 0.86% | 139 | 13.30% | 50 | 2.52% | 20 | 0.96% | 42 | 1.63% | 62 | 2.47% | 325 | 2.71% |
| 302 | 126 | 13.24% | 15 | 1.83% | 77 | 7.37% | 83 | 4.18% | 115 | 5.49% | 231 | 8.94% | 134 | 5.34% | 781 | 6.52% |
| 303 | 5 | 0.53% | 2 | 0.24% | 0 | 0.00% | 17 | 0.86% | 1 | 0.05% | 19 | 0.74% | 42 | 1.67% | 86 | 0.72% |
| 304 | 20 | 2.10% | 110 | 13.45% | 1 | 0.10% | 57 | 2.87% | 12 | 0.57% | 136 | 5.27% | 2 | 0.08% | 338 | 2.82% |
| 305 | 132 | 13.87% | 102 | 12.47% | 98 | 9.38% | 56 | 2.82% | 84 | 4.01% | 123 | 4.76% | 284 | 11.32% | 879 | 7.33% |
| 306 | 24 | 2.52% | 0 | 0.00% | 84 | 8.04% | 0 | 0.00% |  | 0.00% | 190 | 7.36% | 450 | 17.94% | 748 | 6.24% |
| 307 | 12 | 1.26% | 48 | 5.87% | 51 | 4.88% | 32 | 1.61% | 48 | 2.29% | 61 | 2.36% | 153 | 6.10% | 405 | 3.38% |
| **Total** | 952 | 100% | 818 | 100% | 1045 | 100% | 1988 | 100% | 2093 | 100% | 2583 | 100% | 2508 | 100% | 11987 | 100% |
